# Analysis of the *vp*2 gene sequence of a new mutated mink enteritis parvovirus strain in PR China

**DOI:** 10.1186/1743-422X-7-124

**Published:** 2010-06-11

**Authors:** Jing Zuo, Jiahui Rao, Huihui Xu, Liming Ma, Bo Li, Yuping Wang, Xuehui Cai, Wenyu Han, Liancheng Lei, Bin Liu

**Affiliations:** 1 College of Animal Science and Veterinary Medicine JiLin University 5333 Xi 'an Road, Changchun, 130062, China; 2 National Key Laboratory of Veterinary Biotechnology, Harbin Veterinary Research Institute, Chinese Academy of Agricultural Science, 150001, China; 3 Department of Hand Surgery, First Hospital of Jilin University, Changchun, 130021, China

## Abstract

**Background:**

Mink enteritis virus (MEV) causes a highly contagious viral disease of mink with a worldwide distribution. MEV has a linear, single-stranded, negative-sense DNA with a genome length of approximately 5,000 bp. The VP2 protein is the major structural protein of the parvovirus encoded by the *vp*2 gene. VP2 is highly antigenic and plays important roles in determining viral host ranges and tissue tropisms. This study describes the bionomics and *vp*2 gene analysis of a mutated strain, MEV-DL, which was isolated recently in China and outlines its homologous relationships with other selected strains registered in Genbank.

**Results:**

The MEV-DL strain can infect F81 cells with cytopathic effects. Pig erythrocytes were agglutinated by the MEV-DL strain. The generation of MEV-DL in F81 cells could infect mink within three months and cause a disease that was similar to that caused by wild-type MEV. A comparative analysis of the *vp*2 gene nucleotide (nt) sequence of MEV-DL showed that this was more than 99% homologous with other mink enteritis parvoviruses in Genbank. However, the nucleotide residues at positions 1,065 and 1,238 in the MEV-DL strain of the *vp*2 gene differed from those of all the other MEV strains described previously. It is noteworthy that the mutation at the nucleotide residues position 1,238 led to Asp/Gly replacement. This may lead to structural changes. A phylogenetic tree and sequence distance table were obtained, which showed that the MEV-DL and ZYL-1 strains had the closest inheritance distance.

**Conclusions:**

A new variation of the *vp*2 gene exists in the MEV-DL strain, which may lead to structural changes of the VP2 protein. Phylogenetic analysis showed that MEV-DL may originate from the ZYL-1 strain in DaLian.

## Background

Parvoviruses are autonomous, single-stranded DNA viruses that have a genome length of approximately 5,000 bp [1-3]. Parvoviruses are capable of infecting a variety of hosts, for example, parvovirus B19 cause disease in humans, while others such as feline panleukopenia virus (FPV), canine parvovirus (CPV), raccoon parvovirus (RPV) and blue fox parvovirus (BFPV) infect carnivores, and sometimes even fatal to susceptible animals [4-10]. The mink enteritis virus (MEV) disease was first reported by Schofield (1949) [11]. In 1952, Wills [12] isolated and identified the viral pathogen. MEV is a contagious disease can cause acute hemorrhagic enteritis in mink, in particular in younger animals, and it is frequently associated with leucopenia [9].

MEV is classified as a FPV subgroup, a classification which also includes CPV, RPV, and BFPV [13,14]. A comparison of the sequence of amino acid residues between laboratory strains and isolated wild type (wt) strains showed homology of more than 98% [15-21]. The nucleotide sequence of the carnivorous animal parvovirus also has a high level of homology, and the FPV, MEV, RPV, and BFPV cannot be distinguished from each other by DNA sequence alone [22].

VP2 protein is the main structural protein of parvovirus capsid encoded by *vp*2 gene. That either residue 93 or residue 300 in VP2 protein of CPV binds to the cellular transferrin receptor (TfR) determines CPV infectivity of canine [23]. Those capsid regions are also highly antigenic, and serves as the target of many neutralizing antibodies [15].

Tingxiu Jiang [3] reported the first incidence of MEV disease in China in 1981. Nearly 30 years later, this disease has now spread widely across China and affects almost all of the mink cultivation sites [24]. In recent years, although a vaccine has been used to prevent further spread of the disease, the number of infections still continues to grow [24]. This may be related to the capacity of the MEV to continuously mutate. In this study, the MEV-DL virus strain, which is a characteristic parvovirus, has been isolated with a mutation within VP2 protein.

## Methods

### Sample origin and bionomics

Samples of feces from minks with signs of illness were taken from DaLian in China and used for the isolation of MEV-DL. The fecal samples were manipulated according to the methods that have been described previously [25]. The isolated viral particles were then inoculated into F81 cells. When the cytopathic effects of the virus on F81 cells reached 80%, cultures were scraped, then centrifuged after a freeze-thaw cycle twice, and the supernatant was collected [26,27]. Electron microscopy, animal studies [28,29], and hemagglutination tests [23] were used to analyze the bionomics of the MEV-DL strain.

### *vp*2 gene cloning and sequencing

According to the *vp*2 gene sequence published in Genbank (accession number: M23999), a pair of specific primers were designed to amplify the *vp*2 gene of the isolated strain of MEV; the sequence of the forward primer was 5'-GCACCAATGAGTGATGGAGCAGTTC-3' (nt 294-318) and the reverse primer sequence was 5'-TCTAAGGGCAAACCAACCAACCACC-3' (nt 2,292-2,317). The size of the resulting product was 1,999 bp. The fecal samples from the mink that had been infected naturally were homogenized, frozen and thawed in normal saline before being subjected to centrifugation at 4,000 rpm for 20 min. The resulting supernatant was used as the template for PCR, for which the following conditions were applied: 30 cycles of denaturation at 94°C for 1 min, annealing at 55°C for 45 s and polymerization at 72°C for 2 min 30 s. After electrophoresis on a 1.0% agarose gel and ethidium bromide staining, the PCR products were extracted from the gel and puriﬁed. The puriﬁed products were cloned into the PMD18-T vector, transformed into DH-5α, and then incubated at temperature of 37°C for 16 hours. The positive clones were sequenced by Shanghai Sangon Biological Engineering Technology & Services Co., Ltd.

### Phylogeny

Phylogenetic analysis was performed using MEGA 4 [30], and seventeen *vp*2 gene sequences from MEV, CPV and FPV in Genbank were used in this study. A phylogenetic tree was constructed using the neighbor-joining method [31], and a bootstrap analysis with 500 replicates was performed to assess the confidence level of the branch pattern. The sequence distances were determined using the Jotun Hein method [32]. The nt sequences of the *vp*2 gene of the analyzed parvovirus were as follows: MEV-e (U22191), Abashiri (D00765), ZYL-1 (GU272028), MEV-DL (HM015824), Suning (FJ712217), LYT-2 (FJ712221), Beregovoi-Biocentr (AY665656), Rodniki-Biocentr (AY665657), mink enteritis virus (M23999), 389/07 (EU145593), 933/07 (EU360958), ChangC2007 (FJ936171), 04S23 (DQ025992), K029 (EU009205), 128/08 (FJ005246), GR51/08 (GQ865518), 08-5-WH (FJ432717) and 11/09 (GU45715).

## Results

### Bionomics

Sixty hours after inoculation of MEV-DL strain into normal F81 cells, these cells were integrated into a cell colony and cellular strings with the intracellular particles increased. The results from electron microscopy showed that the viral particles existed as a sphere with a diameter of approximately 20 nm. Hemagglutination assays showed that MEV-DL can agglutinate pig erythrocytes. Animal inoculation experiments demonstrated that the MEV-DL cultures infected minks and caused diarrhea 10 days after inoculation; 15 days after inoculation, clinic symptoms of the minks disappeared.

### *vp*2 gene sequencing analysis and phylogenetic analysis

**Figure 1:**
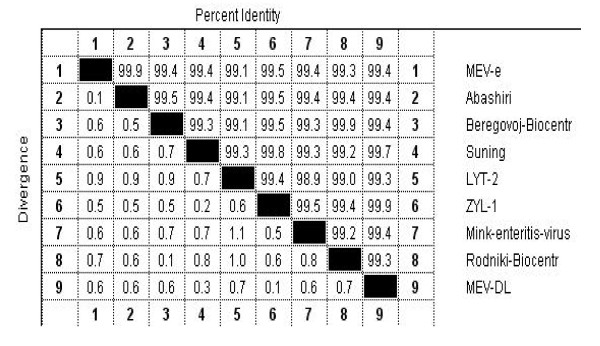
**Divergence and percentage identity of nucleotide sequence variations in the VP2 gene among the nine MEV strains**. a) The VP2 gene sequence of the ZYL-1 strain isolated from DaLian in China (accession number: GU272028) have a homology of up to 99.9% while the ZYT-2 isolation strain (accession number: FJ712221) have a homology of 99.2% when compared with MEV-DL. b) The accession number of the MEV strains shown above are as follows: MEV-e (U22191), Abashiri (D00765), Beregovoi-Biocentr (AY665656), Suning (FJ712217), LYT-2 (FJ712221), ZYL-1 (GU272028), Mink enteritis virus (M23999), Rodniki-Biocentr (AY665657), and MEV-DL (HM015824).

The PCR amplification of the MEV-DL strain *vp*2 gene products were cloned into PMD18-T and sequenced. A comparative analysis of the *vp*2 nucleotide sequence (1,755 bp) of this strain was performed against other MEV *vp*2 sequences that are stored in Genbank. This analysis showed that the mutated strain of MEV-DL was more than 99% homologous with the other strains of MEV cited above ( Figure 1). It was found that there were 33 different nt positions that existed in *vp*2 gene fragments among published *vp*2 sequences of MEV strains. (Additional file 1), but differences occurred at only 16 amino acid residues in the VP2 protein among the strains listed above (Table 1).

**Table 1:** Amino acid and nucleotide sequence variations in the VP2 of nine MEV strains

**VP2**																
Nt position in the alignment	13	88	371	549	694	700	706	882	898/899	1112	1130	1232	1238	1278	1473	1684
aa site in the alignment	5	30	124	183	232	234	236	294	300	371	377	411	413	426	491	562
ZYL-1	A	G	G	M	V	Y	T	L	V	A	R	E	D	N	Q	V
Abashiri	A	G	G	M	I	H	T	L	A	A	R	A	D	N	Q	L
Beregovoi-Biocentr	A	G	G	M	V	H	T	L	L	A	R	A	D	N	Q	V
LYT-2	T	G	G	M	V	H	S	L	I	V	K	E	D	K	Q	V
MEV-e	A	G	G	M	I	H	T	L	A	A	R	E	D	N	H	L
Mink enteritis virus	A	G	G	M	V	Y	T	L	A	A	R	E	D	N	Q	V
Rodniki-Biocentr	A	G	A	M	V	H	T	F	L	A	R	A	D	N	Q	V
Suning	A	R	G	I	V	Y	T	L	V	A	R	E	D	N	Q	V
MEV-DL	A	G	G	M	V	Y	T	L	V	A	R	E	G	N	Q	V

a) In the alignment, the nucleotide (nt) sequence between 1-1,755 corresponds to the VP2 gene and the amino acid (aa) sequence from 1-584. b) Only the mutation at nt position 1,238 led to a Asp/Gly replacement mutation at the 413 aa residue of the MEV-DL VP2 protein, and the LYT-2 strain differed greatly from the other strains shown in the table.

Specifically, the 1,065 and 1,238 nucleotide residues in the *vp*2 gene of the MEV-DL strain differed from those of all MEV strains that have been described previously. It is noteworthy that only the mutation at position 1,238 led to an Asp/Gly replacement mutation at the 413 amino acid residue of the VP2 protein, which may lead to structural changes, such as alterations in the alpha, amphipathic regions and tum regions forecasted by DNASTAR software.

**Figure 2:**
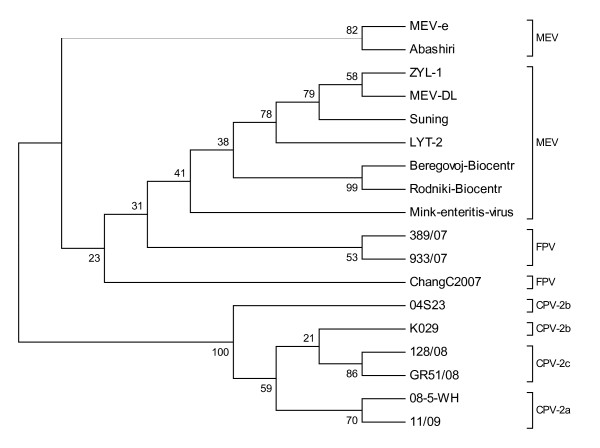
**Phylogenetic analysis based on the complete VP2 nucleotide sequences of different parvovirus isolates**. Nucleotide sequences showed that our MEV-DL isolate was similar to the ZYL-1 and Suning isolates. The sequences of the VP2 genes were obtained from the GenBank. The accession numbers were as follows: MEV-e (U22191), Abashiri (D00765), ZYL-1 (GU272028), MEV-DL (HM015824), Suning (FJ712217), LYT-2 (FJ712221), Beregovoi-Biocentr (AY665656), Rodniki-Biocentr (AY665657), Mink enteritis virus (M23999), 389/07 (EU145593), 933/07 (EU360958), ChangC2007 (FJ936171), 04S23 (DQ025992), K029 (EU009205), 128/08 (FJ005246), GR51/08 (GQ865518), 08-5-WH (FJ432717), and 11/09 (GU45715).

At the phylogenetic level, the *vp*2 gene sequences of the MEV-DL and ZYL-1, as well as the *vp*2 gene sequences of FPV and MEV, formed clusters when compared to the *vp*2 gene sequences of CPV (Figure 2). The results also showed that the *vp*2 gene sequence of the ZYL-1 strain (accession number: GU2772028), which was isolated from DaLian in China, had a nucleotide sequence homology up to 99.9% with the relevant sequence of the MEV-DL strain, whereas the LYT-2 isolation strain (accession number: FJ712221) only had a nucleotide sequence homology up to 99.4% when compared to the MEV-DL strain ( Figure 1).

## Discussion

MEV, FPV, BFPV, RPV and CPV are all classified in the same family and more than 98% of their nucleotide sequences are shared [13]. However, the evolutionary rate of parvoviruses varies between species; for instance, CPV had a nucleotide substitution rate that was similar to that of the RNA viruses, such as HIV, whereas FPV had a slow rate of nt substitution compared to CPV [33]. The average annual replacement rates of CPV and FPV are 1.7×10^-4 ^and 9.4×10^-5^, respectively [33]. In the past 40 years, 33 nucleotide substitution positions have been found in the *vp*2 gene among the MEV strains Mutated bases in the *vp*2 gene in MEV-DL were included in this study. Additionally, a mutated base was also found in BFPV *vp*1 gene at position 296 [34]. Therefore, it can be concluded that more nucleotide substitution positions may exist in the MEV genome than was previously thought, and MEV may have a greater rate of evolution.

Three antigenic variants of MEV have been identified, which differ by only small numbers of amino acid sequence changes in the capsid protein [35]. Cross-immunity to these strains has protected mink from infection by both homologous and heterologous MEV strains [35]. The *vp*2 gene encodes the major structural protein of parvoviruses [36]. It determines the antigenicity of the parvovirus and its host specificity [18]. Therefore, research into the *vp*2 gene is of great interest with regards to vaccine research and viral identification. In our study, analysis of the *vp*2 gene sequence showed that nt residues at positions 1,065 and 1,238 in MEV-DL strain differed from those of all MEV strains described previously. Furthermore, only a mutation at position 1,238 led to an Asp/Gly replacement in the VP2 protein, which is a new variant that has not been reported previously in MEV.

Different parvoviruses show differences in host tropism. For example, FPV can infect mink, but cannot infect canines [37]; likewise, CPV-2a can cause disease in cats, but cannot infect mink [38]. FPV virus could bind with canine transferrin receptors and cause the subsequent infection of canine cells if the 93 and 323 amino acid residues of FPV VP2 protein changed to be the same as that of CPV [39]. In contrast, changes to residues in the vicinity of residue 300 of the amino acid sequence can reduce the amount of adsorption of the virus into canine cells [39]. In the late 1980 s and early 1990 s, the original CPV-2a and CPV-2b strains were replaced by the new CPV-2a and CPV-2b strains, which had resulted from a change in residue 297 of the amino acid sequence of the VP2 protein [40-43].

Sixteen amino acid residues are known to be variable in VP2 proteins of nine MEV strains listed in table 1, and these include in particular the amino acid residues in the vicinity of residue 300 (Table 1). This may lead to changes in MEV-specific adsorption by the host, and further studies should be developed to explain whether the mutation that affects position 413 (Asp/Gly replacement) would lead to changes in host adsorption of MEV and subsequent pathogenicity.

The VP2 protein region (between residues 267 and 498) forms the GH loop located between the βG and βH strands and is affected by the greatest variability among parvoviruses due to its exposure on the capsid surface [44]. In the present study, a strain of MEV has been shown to have a difference at position 413 (Asp/Gly). Residue 413 was not exposed on the capsid surface, as forecasted by DNASTAR, and the change of this position led to alterations in the structure of the alpha, amphipathic and tum regions. This change would be likely to have a significant effect on the structure of the VP2 protein, and so influence its antigenicity and bionomics. This may help to explain the differences between other MEV strains and the variation in response to vaccines that are presently used in China.

In this paper, the *vp*2 nucleotide sequence of the MEV-DL strain has been shown have a homology of up to 99.9% with the ZYL-1 strain (accession number: GU272028). Phylogenetic analysis indicates that only minor changes have occurred between the MEV-DL and ZYL-1 strains. These results infer that the MEV-DL strain may originate from the ZYL-1 strain that was already present in China and, therefore, have little or no relationship to other MEV strains found elsewhere. Due to the high rate of nt substitution in MEV strains, it is necessary to isolate the current MEV strains to understand and prevent the disease caused by MEV.

## Competing interests

The authors declare that they have no competing interests.

## Authors' contributions

L-CL conceived the study, JZ and J-HR planned the experimental aspects of the study, JZ, H-HX and L-MM performed the sequence studies. BL and JZ carried out the cell cultures, and JZ, BL and X-HC contributed to the discussion of all results in this work and drafted the manuscript. W-YH and Y-PW made an equal contribution as L-CL. All of the authors read and approved the final manuscript.
